# Reduced hypothalamic-pituitary-adrenal axis activity in chronic multi-site musculoskeletal pain: partly masked by depressive and anxiety disorders

**DOI:** 10.1186/1471-2474-15-227

**Published:** 2014-07-09

**Authors:** Ellen Generaal, Nicole Vogelzangs, Gary J Macfarlane, Rinie Geenen, Johannes H Smit, Brenda WJH Penninx, Joost Dekker

**Affiliations:** 1 Department of Psychiatry and EMGO Institute for Health and Care Research, VU University Medical Center, PO Box 74077, 1070 BB Amsterdam, The Netherlands; 2 Musculoskeletal Research Collaboration (Epidemiology Group), University of Aberdeen, Aberdeen, UK; 3 Department of Clinical and Health Psychology, Utrecht University, Utrecht, the Netherlands; 4 Department of Rehabilitation Medicine, VU University Medical Center, Amsterdam, The Netherlands

**Keywords:** Hypothalamic-pituitary-adrenal axis, Salivary cortisol, Chronic pain, Central sensitization, Psychopathology

## Abstract

**Background:**

Studies on hypothalamic-pituitary-adrenal axis (HPA-axis) function amongst patients with chronic pain show equivocal results and well-controlled cohort studies are rare in this field. The goal of our study was to examine whether HPA-axis dysfunction is associated with the presence and the severity of chronic multi-site musculoskeletal pain.

**Methods:**

Data are from the Netherlands Study of Depression and Anxiety including 1125 subjects with and without lifetime depressive and anxiety disorders. The Chronic Pain Grade questionnaire was used to determine the presence and severity of chronic multi-site musculoskeletal pain. Subjects were categorized into a chronic multi-site musculoskeletal pain group (n = 471) and a control group (n = 654). Salivary cortisol samples were collected to assess HPA-axis function (awakening level, 1-h awakening response, evening level, diurnal slope and post-dexamethasone level).

**Results:**

In comparison with the control group, subjects with chronic multi-site musculoskeletal pain showed significantly lower cortisol level at awakening, lower evening level and a blunted diurnal slope. Lower cortisol level at awakening and a blunted diurnal slope appeared to be restricted to those without depressive and/or anxiety disorders, who also showed a lower 1-h awakening response.

**Conclusions:**

Our results suggest hypocortisolemia in chronic multi-site musculoskeletal pain. However, if chronic pain is accompanied by a depressive or anxiety disorder, typically related to hypercortisolemia, the association between cortisol levels and chronic multi-site musculoskeletal pain appears to be partly masked. Future studies should take psychopathology into account when examining HPA-axis function in chronic pain.

## Background

Chronic pain, usually defined as pain lasting longer than three months [1], is common with population prevalence estimates of ~10% [2] and results in reduced quality of life [3]. Chronic pain rarely presents at a single body site, it more often presents at multiple body locations, typically in the musculoskeletal system [4-6]. Compared to single-site pain, multi-site musculoskeletal pain has been associated with a greater negative impact on patients’ functioning [7] and disability [6,8], and an increased risk of depressive and anxiety disorders [9]. Whereas acute localized pain is often attributable primarily to damage in the peripheral structures, chronic multi-site musculoskeletal pain can exist without any nociceptive input [1]. Existing interventions of chronic pain are at best moderately effective [10,11] and although epidemiological studies have improved our knowledge on the etiology of chronic pain, its underlying biological mechanisms are still largely unclear.

Evidence suggests that chronic pain conditions may be the consequence of increased neurotransmitter activity of the central nervous system, a process called central sensitization [12]. The hypothalamic-pituitary-adrenal axis (HPA-axis), one of the main bodily stress systems, is likely involved in initiating and perpetuating this process [12,13]. The nature of the relationship between HPA-axis function and chronic pain is however far from fully elucidated [14,15]. A few clinic-based studies (n < 50) in patients with fibromyalgia found hyperactive HPA-axis responses, loss of cortisol diurnal variation with elevated evening cortisol levels [16,17] and increased sensitivity to feedback inhibition of the HPA-axis [18]. Alternatively, two other studies found basal serum cortisol levels in fibromyalgia patients in the normal range [19,20]. However, the majority of previous studies (n < 100; one larger study: n = 429) found reduced activity and impaired feedback sensitivity of the HPA-axis in chronic pain conditions [13,19,21-26], mostly characterized by low basal levels of cortisol as well as a blunted cortisol response to a variety of stressors and dynamic tests. Although evidence is limited, some studies found increased salivary cortisol levels to be associated with higher pain severity scores among subjects with chronic widespread pain [27,28]. However, other studies found decreased cortisol activity and reactivity to be associated with higher pain severity among healthy controls [29] and among chronic pain patients [30]. One prospective study found that subjects with low morning and high evening salivary cortisol levels, indicative of a blunting of the diurnal cortisol rhythm, and increased levels of serum cortisol after the dexamethasone suppression test (DST), indicative of a failure to suppress the HPA-axis, were more likely to develop new-onset chronic widespread pain [24].

Thus, although several studies have suggested a link with HPA-axis dysfunction, whether the HPA-axis is hypoactive or hyperactive in persons with chronic multi-site musculoskeletal pain is yet undetermined. Moreover, most previous studies were rather small in sample size and did not consistently take possible confounders such as lifestyle and disease factors into account. Furthermore, depressive and anxiety disorders have not frequently been considered as covariates that may confound the association between chronic multi-site musculoskeletal pain and HPA-axis function, although these disorders are prevalent among persons with chronic pain and have often been associated with hypercortisolemia [31-34]. Therefore, the present cross-sectional study investigated the hypothesis that HPA-axis dysfunction is associated with the presence and severity of chronic multi-site musculoskeletal pain, while controlling for sociodemographics, lifestyle and disease factors and depressive and anxiety disorders.

## Methods

### Subjects

This cross-sectional study was based on data from the Netherlands Study of Depression and Anxiety (NESDA), an ongoing cohort study conducted among 2981 adults, aged between 18 and 65 years at the baseline assessment. Subjects were recruited from the general population (n = 564), general practices (n = 1610) and from mental health care organizations (n = 807). People in different developmental stages of psychopathology as well as controls with no psychiatric diagnosis participated. The NESDA study contains a high proportion of subjects with chronic multi-site musculoskeletal pain and provides a unique opportunity to control for relevant variables such as depressive and anxiety disorders. Further details of the NESDA study have been described elsewhere [35]. The research protocol was approved by the Central Ethics Committee on Research Involving Human Subjects of the VU University Medical Center Amsterdam and all respondents provided written informed consent.

From the initial 2981 respondents, 767 persons met the criteria for the chronic multi-site musculoskeletal pain group and 887 persons for the control group (criteria discussed in measurements section below). Of these subjects, 455 persons were excluded because no valid salivary cortisol values were available and 74 persons because they used corticosteroids that are known to influence HPA-axis function. There were no pregnant or breastfeeding women in our study sample, leaving a total of 1125 subjects for our analyses (471 with chronic multi-site musculoskeletal pain and 654 controls).

Excluded subjects (n = 529) were significantly younger (40.2 *vs.* 44.7 years, p < 0.001), had fewer years of education (11.6 *vs.* 12.4 years, p < 0.001), had more often chronic multi-site musculoskeletal pain (56.0 *vs.* 41.9%, p < 0.001) and had more often a lifetime depressive and/or anxiety disorder (80.2 *vs.* 71.2%, p < 0.001), but did not differ in sex, compared with included subjects (n = 1125). Differences in covariates between excluded and included subjects were similar within the chronic pain group and, apart from that, in the control group.

### Chronic multi-site musculoskeletal pain

Chronic multi-site musculoskeletal pain was measured using the Chronic Pain Grade (CPG) [36], a reliable and valid measure of severity of chronic pain [37,38]. The CPG first inquires about the presence of pain in the prior six months on several locations (*i.e.*, the extremities [joints of the arms, hands, legs or feet], back, neck, head, abdomen, chest, and the mouth and face [orofacial area]) [39]. The subsequent questions in the CPG refer to the most painful location and include: 1) days in pain in the prior six months (scale 0-180); 2) pain at this moment (scale 0-10); 3) worst pain in the prior six months (scale 0-10); 4) average pain in the prior six months (scale 0-10); 5) disability days in the prior six months (scale 0-180); 6) disability in daily activities (scale 0-10); 7) disability in spare time, social life, and family activities (scale 0-10); and 8) disability in work (scale 0-10). According to the CPG protocol, five grades were categorized from these measures: grade 0 (pain free, no pain in the prior six months); grade I (low disability, low intensity); grade II (low disability, high intensity); grade III (high disability, moderately limiting); and grade IV (high disability, severely limiting).

The CPG was used to classify two groups. Subjects were classified as having chronic multi-site musculoskeletal pain if they had grade I, II, III or IV and pain present in (one or more of) the extremities, and the back and the neck (n = 471). For reasons of clarity, we further refer to the chronic multi-site musculoskeletal pain group as the chronic pain group. The control group consisted of people with grade 0 (n = 130) or with grade I and pain in the prior six months in at most 2 locations (n = 524). The remaining subjects who did not meet the criteria of the chronic pain group or the control group were not eligible to be included in the present study (n = 1327). Of the total NESDA sample, approximately 26% met criteria for chronic multi-site musculoskeletal pain.

For examination of pain severity, pain intensity and pain disability were assessed in subjects with chronic pain. For assessment of pain intensity, questions 2, 3 and 4 of the CPG were used (see above) to yield a total pain intensity score (average of the 0-10 ratings of the 3 questions multiplied by 10 resulting in a 0-100 score) [36]. For assessment of pain disability, questions 6, 7 and 8 of the CPG were used (see above) to yield a total pain disability score (average of the 0-10 ratings of the 3 questions multiplied by 10 resulting in a 0-100 score) [36].

### HPA-axis function

As described in more detail elsewhere [40], respondents collected saliva samples at home on a (regular) working day shortly after the baseline interview. The median time between the interview and saliva sampling was 9.0 days (IQR: 5-22). Instructions prohibited eating, smoking, drinking, or brushing teeth within 15 minutes before sampling. Saliva samples were obtained using Salivettes (Sarstedt AG and Co, Numbrecht, Germany) at seven time points covering 1-h awakening cortisol, evening values and a dexamethasone suppression test. The cortisol awakening response (CAR) includes four sampling points: at awakening (T1) and 30 (T2), 45 (T3), and 60 (T4) minutes later. Two evening values were collected at 10 p.m. (T5) and 11 p.m. (T6). Dexamethasone suppression was measured by cortisol sampling the next morning on awakening (T7) after ingestion of 0.5 mg dexamethasone, directly after the saliva sample at 11 p.m. (T6) was taken. Samples were stored in refrigerators and returned by mail. Cortisol analysis was performed by competitive electrochemiluminescence immunoassay (E170 Roche, Switzerland). The functional detection limit was 2.0 nmol/l and the intra- and interassay variability coefficients in the measuring range were less than 10%.

The first cortisol value directly after awakening on the first day (T1) was considered as a basal indicator of cortisol level. In addition, using formulas described by Pruessner *et al.*[41], the area under the curve with respect to the ground (AUCg) and with respect to the increase (AUCi) were calculated based on the four morning cortisol measures. The AUCg is an estimate of the total cortisol secretion over the first hour of awakening; the AUCi is a measure of the dynamics of the cortisol awakening response related to the sensitivity of the system and emphasizing the rate of change of the cortisol levels after awakening.

Evening cortisol was assessed as another basal indicator of cortisol level. The mean of the two evening values (at 10 p.m. and 11 p.m.) was used to reflect mean evening cortisol; the correlation between the two evening values was *r* = 0.55.

Diurnal slope was calculated by subtracting the value at 11 p.m. (T6) from the sample at awakening (T1) and dividing it by the number of hours in-between the two samples, resulting in the decline in cortisol level per hour. If the evening value at T6 was missing, the evening value at 10 P.M. (T5) was used if available (n = 12).

The cortisol suppression ratio was calculated by the cortisol value at awakening on the first day (T1) divided by the cortisol value at awakening on the next day (T7) after ingestion of 0.5 mg dexamethasone the evening before.

### Covariates

First, cortisol sampling characteristics were considered because these have been associated with salivary cortisol in NESDA [40]. The sampling factors comprised awakening time, working on day of sampling (yes/no), month of sampling (light months March through September *vs.* dark months October through February), and ≤6 h sleep (yes/no). In addition, basic potential confounders included sociodemographic characteristics (age, sex, and years of education).

A second set of covariates included lifestyle and disease factors as these have been previously associated with both HPA-axis function and chronic pain. Body mass index was calculated as weight in kilograms divided by height in meters squared. Smoking was categorized as never smoker, former smoker and current smoker. Alcohol use was categorized as non-drinker, mild/moderate drinker (1–21 glasses/week for men and 1–14 glasses/week for women), and heavy drinker (>21 glasses/week for men and >14 glasses/week for women) [42]. Physical activity was assessed with the International Physical Activity Questionnaire [43] and expressed in 1000 metabolic equivalent minutes per week. Number of chronic diseases (including cardiovascular disease, epilepsy, diabetes mellitus, osteoarthritis, stroke, cancer, chronic lung disease, thyroid disease, liver disease, intestinal disorders, and ulcers) were assessed by self-report. Chronic diseases were considered present if persons had received treatment.

A third set of covariates included lifetime diagnoses of depressive and/or anxiety disorders and use of antidepressants. Lifetime diagnoses included both current (*i.e.* in past six months) and prior diagnoses of depressive disorders (major depressive disorder and dysthymia) and anxiety disorders (panic disorder, agoraphobia, generalized anxiety disorder and social phobia) and were established with the Composite International Diagnostic Interview (WHO version 2.1), a widely accepted and reliable instrument that assesses mental disorders according to DSM standards [44]. Medication use was assessed based on drug container inspection of all drugs used in the past month and classified according to the World Health Organization (WHO) Anatomical Therapeutic Chemical (ATC) classification [45]. Use of antidepressants were considered because of their reported association with cortisol level [46] and pain level [47]. Antidepressant medication included tricyclic antidepressants [TCA; ATC code N06AA], selective serotonin reuptake inhibitors [SSRI; ATC code N06AB] and other antidepressant medications [ATC codes N06AF/AG/AX]) and were considered if frequently used (daily or >50% of the time).

### Statistical analyses

Descriptive baseline characteristics were reported as mean, median, or percentage across persons with chronic pain and controls. Differences between groups were examined using independent-sample t tests for continuous variables, chi-square tests for dichotomous and categorical variables, and Mann-Whitney U tests for non-normally distributed variables.

Logistic regression analyses were performed to analyze the association between cortisol measures and the presence of chronic pain (*vs.* controls). Also, a U-curved association of cortisol with the presence of chronic pain was considered by entering quadratic terms of cortisol measures to the model also including the linear terms. To examine whether cortisol measures were associated with pain severity in subjects with chronic pain, separate linear regression analyses were conducted with cortisol measures as predictors and pain intensity and pain disability as outcome variables.

In all logistic and linear regression analyses, three models were tested: (1) adjusted for cortisol sampling factors and sociodemographic variables; (2) additionally adjusted for lifestyle and disease factors, and; (3) additionally adjusted for lifetime diagnoses of depressive or anxiety disorders and use of antidepressants.

Because depressive and anxiety disorders have previously been associated with hypercortisolemia [31], we subsequently examined the association between cortisol and chronic pain, free from any depression/anxiety effects. Therefore, we tested the possible moderating effect of depressive and/or anxiety disorders on the association between cortisol and chronic pain. Chronic pain subjects without and chronic pain subjects with lifetime depressive and anxiety disorders (LDA) were each compared with control subjects without chronic pain and without LDA by performing adjusted multinomial logistic regression analyses.

For all statistical tests, a probability level of less than or equal to 5% was regarded as significant. The statistical calculations were performed using SPSS, version 20.0.

## Results

### Baseline characteristics

Baseline characteristics of the study population are shown in Table 1. In subjects with chronic multi-site musculoskeletal pain, the mean days of pain in the prior six months was 109.0 (SD = 69.9), the mean pain intensity score was 52.3 (SD = 17.7) and the mean pain disability score was 38.1 (SD = 24.9). Compared to controls, subjects with chronic pain differed in all cortisol sampling factors, were significantly older, were more often women, had less education, had a higher body mass index, were more often current smokers, were more often non-drinkers, had more chronic diseases, had more often a current and a lifetime comorbid depressive and/or anxiety disorder, and used more often antidepressants (all p < 0.05). Regarding cortisol measures, diurnal slope was significantly lower in the chronic pain group in comparison to the control group. For the other cortisol measures, differences between subjects with and without chronic pain were not significant in unadjusted analyses.

**Table 1:** Baseline characteristics*

	**Controls**	**Chronic pain**	***P*** **†**	***N***^**~**^
	**(n = 654)**	**(n = 471)**		
**Pain**				
Days of pain in the prior 6 months	28.3 ± 49.1	109.0 ± 69.9	<0.001	
Pain intensity	21.5 ± 14.7	52.3 ± 17.7	<0.001	
Pain disability	9.4 ± 13.2	38.1 ± 24.9	<0.001	
**Cortisol sampling factors**				
Time of awakening	7:23 ± 1:07	7:34 ± 1:10	0.01	
Working on day of sampling, %	67.2	48.5	<0.001	
Sampling in month with more daylight, %	52.7	54.4	0.50	
≤6 h sleep, %	21.0	37.3	<0.001	
**Sociodemographic factors**				
Age, years	43.3 ± 13.4	46.7 ± 11.8	<0.001	
Women, %	56.7	73.5	<0.001	
Education, years	13.1 ± 3.3	11.6 ± 3.3	<0.001	
**Lifestyle and disease factors**				
Body mass index, kg/m^2^	25.0 ± 4.2	26.2 ± 4.8	<0.001	
Smoking, %			0.05	
Never smoker	32.0	27.2		
Former smoker	39.0	37.2		
Current smoker	29.1	35.7		
Alcohol use, %			<0.001	
Non-drinker	20.9	38.2		
Mild/moderate drinker	66.2	50.7		
Heavy drinker	12.8	11.0		
Physical activity, 1000 MET min./week	3.6 ± 3.0	3.9 ± 3.1	0.06	
Number of chronic diseases	0.6 ± 0.8	0.9 ± 0.9	<0.001	
**Depression and anxiety factors**				
Lifetime depressive or anxiety disorder, %			<0.001	
No disorder	40.4	12.7		
Depressive disorder	19.0	16.8		
Anxiety disorder	13.1	10.0		
Comorbid depressive/anxiety disorder	27.5	60.5		
Current depressive or anxiety disorder, %	35.9	67.5	<0.001	
Antidepressant medication, %			<0.001	
No antidepressant	83.2	71.3		
SSRI	10.9	20.0		
TCA	2.1	2.3		
Other antidepressant	3.8	6.4		
**Cortisol measures**				
At awakening, nmol/l, median [IQR]	15.88 [12.42-20.17]	15.48 [12.07-19.45]	0.10	1105
AUCg, nmol/l/h	19.0 ± 7.0	18.8 ± 6.7	0.55	1025
AUCi, nmol/l/h	1.9 ± 6.5	2.6 ± 6.2	0.09	1025
Mean evening level, nmol/l, median [IQR]	4.68 [3.23-6.34]	4.90 [3.38-6.83]	0.07	1120
Diurnal slope, decline in nmol/l/h	0.77 ± 0.49	0.71 ± 0.39	0.05	1001
Cortisol suppression ratio, median [IQR]	2.43 [1.75-3.33]	2.35 [1.70-3.12]	0.23	1061

MET = metabolic equivalent; SSRI = selective serotonin reuptake inhibitor; TCA = tricyclic antidepressant.

AUCg = area under the curve with respect to the ground; AUCi = area under the curve with respect to the increase.

*Values are mean ± SD unless otherwise indicated. †Based on independent-sample T test for continuous

variables, chi-square test for dichotomous and categorical variables and Mann-Whitney *U* test for non-normally distributed variables. ^***~***^N-values that vary from total N = 1125 due to missings on individual cortisol measures.

### HPA-axis function and the presence and severity of chronic multi-site musculoskeletal pain

Table 2 reports the results of the adjusted logistic regression analyses, assessing the association between cortisol measures and the presence of chronic multi-site musculoskeletal pain. Lower cortisol levels at awakening were associated with the presence of chronic pain after adjustment for confounders. Lower evening cortisol levels were associated with the presence of chronic pain after adjustment for lifestyle and disease factors and after full adjustment for depression and anxiety factors. The association between a flatter diurnal slope and the presence of chronic pain was not significant after sociodemographic adjustment and after additional adjustment for lifestyle and disease factors, but this association became statistically significant after adjustment for depression and anxiety factors. For AUCg, AUCi and cortisol suppression ratio, no statistically significant associations with the presence of chronic pain were found.

**Table 2:** Associations* between cortisol measures and the presence of chronic multi-site musculoskeletal pain (n = 1125)

	**OR (95% CI)**	***P***
**At awakening**		
Sociodemographic^a^	0.86 (0.75, 0.98)	0.03
Lifestyle & disease^b^	0.83 (0.72, 0.95)	0.009
Depression and anxiety^c^	0.81 (0.70, 0.94)	0.006
**AUCg**		
Sociodemographic^a^	0.94 (0.83, 1.08)	0.40
Lifestyle & disease^b^	0.91 (0.79, 1.06)	0.23
Depression and anxiety^c^	0.89 (0.76, 1.04)	0.13
**AUCi**		
Sociodemographic^a^	1.10 (0.97, 1.26)	0.15
Lifestyle & disease^b^	1.12 (0.97, 1.30)	0.12
Depression and anxiety^c^	1.10 (0.94, 1.28)	0.25
**Mean evening level**		
Sociodemographic^a^	0.92 (0.80, 1.05)	0.21
Lifestyle & disease^b^	0.85 (0.72, 0.99)	0.04
Depression and anxiety^c^	0.85 (0.72, 1.00)	0.05
**Diurnal slope**		
Sociodemographic^a^	0.89 (0.77, 1.02)	0.10
Lifestyle & disease^b^	0.88 (0.76, 1.02)	0.10
Depression and anxiety^c^	0.85 (0.73, 0.99)	0.04
**Cortisol suppression ratio**		
Sociodemographic^a^	1.02 (0.89, 1.16)	0.80
Lifestyle & disease^b^	1.03 (0.90, 1.18)	0.69
Depression and anxiety^c^	1.04 (0.90, 1.20)	0.57

*Based on adjusted logistic regression analyses; OR per 1 SD increase;

At awakening: SD = 6.78, AUCg: SD = 6.87, AUCi: SD = 6.39, mean evening level: SD = 3.43, diurnal slope: SD = 0.45, cortisol suppression ratio: SD = 1.58.

^a^Adjusted for awakening time, working on day of sampling, month of sampling, ≤6 hours of sleep, age, sex, and years of education.

^b^Additionally adjusted for body mass index, smoking, alcohol intake, physical activity, and chronic diseases; ^c^additionally adjusted for lifetime depressive/anxiety disorder and antidepressants.

To test for a possible U-curved association with the presence of chronic pain, quadratic terms of cortisol measures were examined. These adjusted logistic regression analyses revealed that the quadratic term of diurnal slope was significantly associated with the presence of chronic pain (p = 0.05; all other p ≥ .15). Therefore, quintiles of diurnal slope were created and entered as predictors in adjusted logistic regression analyses with chronic pain as outcome. No evidence was found for a U-curved association when the lowest and highest quintile of diurnal slope were compared with intermediate categories (lowest quintile: OR = 1.09, p = 0.65; highest quintile: OR = 0.72, p = 0.08) (data not shown).

Table 3 reports the associations between cortisol measures and pain intensity and pain disability among subjects with chronic pain (n = 471). No significant associations were found. Additional analyses considering chronic pain subjects without lifetime depression and anxiety also indicated no associations for cortisol with pain severity (data of additional analyses not tabulated).

**Table 3:** Associations* between cortisol measures and pain intensity and pain disability in subjects with chronic multi-site musculoskeletal pain (n = 471)

	**Pain intensity**		**Pain disability**	
	**Beta**	***P***	**Beta**	***P***
**At awakening**				
Sociodemographic^a^	0.05	0.32	0.01	0.86
Lifestyle & disease^b^	0.04	0.42	0.00	0.94
Depression and anxiety^c^	0.02	0.72	-0.01	0.86
**AUCg**				
Sociodemographic^a^	0.01	0.86	-0.05	0.28
Lifestyle & disease^b^	-0.01	0.92	-0.05	0.34
Depression and anxiety^c^	-0.03	0.54	-0.06	0.22
**AUCi**				
Sociodemographic^a^	-0.05	0.32	-0.06	0.21
Lifestyle & disease^b^	-0.06	0.18	-0.06	0.23
Depression and anxiety^c^	-0.07	0.15	-0.06	0.23
**Mean evening level**				
Sociodemographic^a^	0.00	0.99	-0.03	0.59
Lifestyle & disease^b^	-0.01	0.80	-0.01	0.81
Depression and anxiety^c^	-0.01	0.79	-0.01	0.93
**Diurnal slope**				
Sociodemographic^a^	0.06	0.21	0.03	0.61
Lifestyle & disease^b^	0.05	0.29	0.01	0.78
Depression and anxiety^c^	0.03	0.57	0.00	0.94
**Cortisol suppression ratio**				
Sociodemographic^a^	0.00	0.98	0.02	0.68
Lifestyle & disease^b^	0.01	0.82	0.02	0.67
Depression and anxiety^c^	0.01	0.89	0.01	0.76

*Based on adjusted linear regression analyses; ^a^adjusted for awakening time, working on day of sampling, month of sampling, ≤6 hours of sleep, age, sex, and years of education; ^b^additionally adjusted for body mass index, smoking, alcohol intake, physical activity, and chronic diseases; ^c^additionally adjusted for lifetime depressive/anxiety disorder and antidepressants.

### HPA-axis function and chronic multi-site musculoskeletal pain without/with depression and anxiety

This study found hypoactive HPA-axis function in chronic multi-site musculoskeletal pain (Table 2), while depressive and some types of anxiety disorders have previously been associated with hypercortisolemia [31,32], also in the NESDA study [33,34]. To further examine possible moderating effects, we subsequently tested whether depressive and/or anxiety disorders mask the association between cortisol measures and the presence of chronic pain. Adjusted multinomial logistic regression analyses were performed for chronic pain subjects without lifetime depressive and/or anxiety disorders (n = 60) and chronic pain subjects with lifetime depressive and/or anxiety disorders (n = 411) separately compared to control subjects (without chronic pain and without lifetime depressive and/or anxiety disorders; n = 264) (see Table 4). These analyses indicated that lower cortisol at awakening, lower AUCg and a flatter diurnal slope were significantly associated with higher odds of chronic pain in persons without depressive and anxiety disorders. Additional adjustment for lifestyle and disease factors hardly affected these results. In contrast, none of the cortisol measures were significantly associated with chronic pain with depressive and/or anxiety disorders. When directly comparing chronic pain subjects without depressive and/or anxiety disorders to chronic pain subjects with depressive and/or anxiety disorders, a significant difference for cortisol at awakening was found after sociodemographic adjustment. This difference was only marginally significant after adjustment for lifestyle and disease. In addition, AUCg and diurnal slope significantly differed between chronic pain subjects without and with depressive and/or anxiety disorders before and after adjustment for confounders.

**Table 4:** Associations* between cortisol measures and the presence of chronic multi-site musculoskeletal pain in persons without and with a lifetime depressive and/or anxiety disorder

	**Chronic pain without LDA (n = 60) *vs. *** **controls (n = 264)**		**Chronic pain with LDA (n = 411) *vs. *** **controls (n = 264)**		**Chronic pain with LDA *vs. *** **chronic pain without LDA**^**c**^
	**OR (95% CI)**	***P***	**OR (95% CI)**	***P***	***P***
**At awakening**					
Sociodemographic^a^	0.67 (0.47, 0.94)	0.02	0.94 (0.79, 1.12)	0.50	0.04
Lifestyle & disease^b^	0.66 (0.46, 0.93)	0.02	0.90 (0.74, 1.09)	0.27	0.07
**AUCg**					
Sociodemographic^a^	0.61 (0.43, 0.89)	0.009	1.04 (0.86, 1.25)	0.68	0.003
Lifestyle & disease^b^	0.62 (0.42, 0.90)	0.01	0.96 (0.79, 1.18)	0.72	0.02
**AUCi**					
Sociodemographic^a^	0.95 (0.69, 1.32)	0.77	1.15 (0.96, 1.38)	0.14	0.24
Lifestyle & disease^b^	1.00 (0.71, 1.40)	0.99	1.11 (0.91, 1.36)	0.30	0.51
**Mean evening level**					
Sociodemographic^a^	0.89 (0.61, 1.29)	0.53	0.96 (0.79, 1.17)	0.68	0.67
Lifestyle & disease^b^	0.93 (0.64, 1.33)	0.68	0.82 (0.66, 1.02)	0.07	0.49
**Diurnal slope**					
Sociodemographic^a^	0.71 (0.49, 1.01)	0.06	1.03 (0.85, 1.24)	0.80	0.03
Lifestyle & disease^b^	0.70 (0.49, 0.99)	0.05	1.04 (0.85, 1.27)	0.69	0.02
**Cortisol suppression ratio**					
Sociodemographic^a^	0.89 (0.65, 1.21)	0.44	0.95 (0.81, 1.11)	0.51	0.67
Lifestyle & disease^b^	0.86 (0.62, 1.19)	0.36	0.99 (0.83, 1.17)	0.87	0.40

LDA = Lifetime depressive and/or anxiety disorder.

*Based on adjusted multinomial logistic regression analyses using persons without chronic pain and without LDA as the reference group (n = 264); OR per 1 SD increase; At awakening: SD = 6.78, AUCg: SD = 6.87, AUCi: SD = 6.39, mean evening level: SD = 3.43, diurnal slope: SD = 0.45, cortisol suppression ratio: SD = 1.58.

^a^Adjusted for awakening time, working on day of sampling, month of sampling, ≤6 hours of sleep, age, sex, years of education; ^b^additionally adjusted for body mass index, smoking, alcohol intake, physical activity, and chronic diseases; ^c^based on the same multinomial logistic regression analyses using chronic pain without LDA as the reference group.

## Discussion

The main objective of the current cross-sectional study was to investigate the association between HPA-axis dysregulation and the presence and severity of chronic multi-site musculoskeletal pain. Chronic pain subjects in this study show lower awakening cortisol levels, lower evening levels and a blunted diurnal slope compared to controls. These associations appeared to be partly masked by comorbid depressive and/or anxiety disorders, which are typically accompanied by hypercortisolemia.

Our findings of hypocortisolemia and a blunted diurnal cortisol slope in subjects with chronic multi-site musculoskeletal pain are in line with other studies that reported a hypoactive HPA-axis in chronic pain conditions [13,19,21-24]. This blunting of the HPA-axis has been proposed to occur after prolonged exposure to stress [14]. During acute stress, the adrenal cortex produces more cortisol which in turn inhibits the secretion of corticotrophin-releasing hormone (CRH) from the hypothalamus and adrenocorticotropic hormone (ACTH) from the pituitary to eventually normalize cortisol release and maintain body homeostasis [14]. Before the development of chronic pain, the HPA-axis may start hyperactive, but after long-term hyperactivity the stress system may reach a state of exhaustion and the HPA-axis turns to a state of hypoactivity [48]. This hypoactive state of the HPA-axis is also suggested by hyperreactivity of ACTH after CRH infusion suggesting up-regulation of pituitary CRH receptors due to a hypoactive state of endogeneous CRH and hypocortisolemia [24,49,50].

Morning hypocortisolemia and a blunted diurnal cortisol slope were most clearly present in chronic pain subjects without lifetime depressive and/or anxiety disorders. Depression and specific types of anxiety disorders have often been associated with hypercortisolemia [31,32,51], as also previously demonstrated in the NESDA study (*i.e.*, increased cortisol awakening response for both current and remitted major depressive disorder and panic disorder with agoraphobia [33,34]). Several other studies outlined a distinction between HPA-axis hyperactivity in depression and HPA-axis hypoactivity in chronic pain [13,52], which suggests distinct biological processes in psychological disorders and pain conditions. Additional analyses in our sample indicate that both a current diagnosis and a prior history of depressive and/or anxiety disorders mask hypocortisolemia in chronic multi-site musculoskeletal pain. Altered morning cortisol level may represent a trait rather than a state which reflects biological vulnerability or a biological scar resulting from depression and anxiety [34].

Although the current study did not measure nocturnal cortisol levels, hypocortisolemia at awakening might indicate reduced cortisol secretion during the late phase of sleep or differences in frequency of cortisol peaks overnight [17,53]. A previous longitudinal study found non-restorative sleep (waking feeling unrefreshed) to be associated with the resolution of chronic widespread pain [54]. However, the role of sleep-related disturbances on HPA-axis perturbations in chronic multi-site musculoskeletal pain warrants further research.

We found lower evening cortisol levels in the overall sample of subjects with chronic multi-site musculoskeletal pain compared to controls, which is in contrast with elevated evening levels found in previous studies in patients with fibromyalgia with a smaller sample size than the current study [16,17]. However, in support of our findings, a cross-sectional study by McBeth *et al.* showed that a lower saliva cortisol score, composed of morning and evening cortisol levels, was associated with chronic widespread pain and being psychologically at risk of chronic widespread pain [25].

No significant associations with chronic multi-site musculoskeletal pain were found for the dynamic of the cortisol awakening response (AUCi) and post-dexamethasone cortisol levels, which is in contrast to a previous study that demonstrated non-suppression of the HPA-axis in 131 subjects with chronic widespread pain by elevated serum cortisol levels after a 0.25 mg dexamethasone suppression test [25]. This opposite finding might be explained by heterogeneity of samples or measurement differences, *e.g.* in the assessment of pain, depression, anxiety and cortisol [55]. Moreover, as also suggested by the results of our study, adjusting for the effects of psychological distress might attenuate the association between post-stress cortisol levels and chronic multi-site musculoskeletal pain [25].

A previous study found that hypocortisolemia was present in women with fibromyalgia but not in men [56]. Our study could not examine gender differences in the association between cortisol and chronic pain, free of the effects of psychopathology, because group sample sizes would become too small.

In contrast to the hypothesis that HPA-axis dysregulation was associated with pain severity, no associations with HPA-axis function were found for both pain intensity and pain disability among persons with chronic multi-site musculoskeletal pain. This might suggest that once hypocortisolemia is present in subjects with chronic multi-site musculoskeletal pain, hypoactivity of the HPA-axis does not further impact pain severity. Prospective studies should further investigate the possible differential role for hypocortisolemia in the onset and perpetuation of chronic multi-site musculoskeletal pain.

Several methodological issues related to this study should be noted. First, our cross-sectional study design does not allow to draw conclusions regarding causality. Longitudinal studies are needed to examine whether HPA-axis hypoactivity is the cause or the consequence of chronic multi-site musculoskeletal pain and its correlates such as sleep disturbance, inactivity, and low fitness. Second, previous studies mostly examined chronic widespread pain using diagnostic criteria by Wolfe *et al.*[57] in which participants need to have axial and bilateral pain above and below the waist. To best reflect this definition of axial and bilateral pain [57], we classified persons as having chronic multi-site musculoskeletal pain if they had pain in (one or more of) the extremities, the back AND the neck. Using our criteria of chronic pain, we may have included patients with milder pain complaints than previous studies that investigated fibromyalgia patients with chronic widespread pain in all four body quadrants. However, chronic multi-site pain can occur without meeting the classification criteria for chronic widespread pain and therefore setting broader parameters for studying biological mechanisms in chronic pain patients might be useful [4,5]. Third, chronic pain is defined as pain for ≥3 months, whereas we used the CPG that assesses the number of days in pain in the prior 6 months. Our subjects with chronic multi-site musculoskeletal pain had on average 109 days of pain (~3.5 months) in the most painful location in the prior 6 months. Additional analyses considering only chronic pain subjects with pain for ≥3 months showed similar odds ratio’s for cortisol at awakening, AUCg and diurnal slope (as compared to Table 4). Therefore, it appears unlikely that the criterion to define chronic pain explains our findings. Fourth, to increase sample size, we included persons with grade I on the CPG and pain in ≤2 locations in the control group. Comparisons with a more strict control group might elucidate even stronger associations. However, chronic pain in 1 or 2 locations is quite common in the population [2] which suggests that our control group resembled the general population and even improved the generalizability of our findings. Fifth, the ambulatory cortisol measures used in our study might not have been as rigorous as those in laboratory settings, because we did not have full control over the sample collection and the ingestion of the 0.5 mg dexamethasone. However, in a pilot study among 47 respondents, 90% showed detectable dexamethasone levels in the saliva sample the next morning at awakening [40] suggesting that non-compliance with dexamethasone ingestion is unlikely to explain our findings. Finally, HPA-axis function fluctuates from day-to-day while we only sampled cortisol on one day. Although the median time lap between the baseline interview and cortisol sampling was only 9 (IQR: 5-22) days, subjects did not report current mood or other state effects on the day of sampling. Nonetheless, we adjusted for current mood disorders in our analyses, which are highly correlated with current mood. This study also has clear strengths as compared to previous studies in the field, including its large sample size, a detailed assessment of HPA-axis functioning and the possibility to control for various confounders. Because a large part of this study sample consisted of persons with depressive and/or anxiety disorders, the unique possibility was provided to closely examine the association between HPA-axis function and chronic multi-site musculoskeletal pain while taking into account the possible effects of depressive and anxiety disorders which are often present in persons with chronic pain [34,40].

## Conclusions

The present study indicates hypocortisolemia in chronic multi-site musculoskeletal pain, which appears to be partly masked by the presence of comorbid depressive and/or anxiety disorders. Future studies should take psychopathology into account when examining HPA-axis function in chronic multi-site musculoskeletal pain. However, whether hypocortisolemia is also associated with the onset or perpetuation of chronic multi-site musculoskeletal pain should be further examined in longitudinal analyses.

## Competing interests

The authors declare that they have no competing interests.

## Authors’ contributions

Authors’ Dr. BP and Dr. JD were involved in overall study design and funding. Authors EG, Dr. JD, Dr. BP and Dr. NV designed and wrote the analysis plan for the current paper. Author EG undertook the statistical analyses. All authors were involved in the interpretation of data. Author EG wrote the first draft of the manuscript. All authors critically read the manuscript to improve intellectual content. All authors have approved the final manuscript in its present form.

## Pre-publication history

The pre-publication history for this paper can be accessed here:

http://www.biomedcentral.com/1471-2474/15/227/prepub
